# Understanding the Subjective Experience of Long-term Remote Measurement Technology Use for Symptom Tracking in People With Depression: Multisite Longitudinal Qualitative Analysis

**DOI:** 10.2196/39479

**Published:** 2023-01-26

**Authors:** Katie M White, Erin Dawe-Lane, Sara Siddi, Femke Lamers, Sara Simblett, Gemma Riquelme Alacid, Alina Ivan, Inez Myin-Germeys, Josep Maria Haro, Carolin Oetzmann, Priya Popat, Aki Rintala, Elena Rubio-Abadal, Til Wykes, Claire Henderson, Matthew Hotopf, Faith Matcham

**Affiliations:** 1 Department of Psychological Medicine King's College London London United Kingdom; 2 Department of Psychology King's College London London United Kingdom; 3 Parc Sanitari Sant Joan de Déu, Fundació Sant Joan de Déu CIBERSAM Universitat de Barcelona Barcelona Spain; 4 Department of Psychiatry Amsterdam University Medical Center Vrije Universiteit Amsterdam Netherlands; 5 Center for Contextual Psychiatry Department of Neurosciences UK Leuven Leuven Belgium; 6 Health Service & Population Research Department King's College London London United Kingdom; 7 School of Psychology, University of Sussex Falmer Sussex United Kingdom

**Keywords:** remote measurement, technology, qualitative, engagement, telehealth, depression, mental health, mobile phone

## Abstract

**Background:**

Remote measurement technologies (RMTs) have the potential to revolutionize major depressive disorder (MDD) disease management by offering the ability to assess, monitor, and predict symptom changes. However, the promise of RMT data depends heavily on sustained user engagement over extended periods. In this paper, we report a longitudinal qualitative study of the subjective experience of people with MDD engaging with RMTs to provide insight into system usability and user experience and to provide the basis for future promotion of RMT use in research and clinical practice.

**Objective:**

We aimed to understand the subjective experience of long-term engagement with RMTs using qualitative data collected in a longitudinal study of RMTs for monitoring MDD. The objectives were to explore the key themes associated with long-term RMT use and to identify recommendations for future system engagement.

**Methods:**

In this multisite, longitudinal qualitative research study, 124 semistructured interviews were conducted with 99 participants across the United Kingdom, Spain, and the Netherlands at 3-month, 12-month, and 24-month time points during a study exploring RMT use (the Remote Assessment of Disease and Relapse-Major Depressive Disorder study). Data were analyzed using thematic analysis, and interviews were audio recorded, transcribed, and coded in the native language, with the resulting quotes translated into English.

**Results:**

There were 5 main themes regarding the subjective experience of long-term RMT use: research-related factors, the utility of RMTs for self-management, technology-related factors, clinical factors, and system amendments and additions.

**Conclusions:**

The subjective experience of long-term RMT use can be considered from 2 main perspectives: experiential factors (how participants construct their experience of engaging with RMTs) and system-related factors (direct engagement with the technologies). A set of recommendations based on these strands are proposed for both future research and the real-world implementation of RMTs into clinical practice. Future exploration of experiential engagement with RMTs will be key to the successful use of RMTs in clinical care.

## Introduction

### Background

Depressive disorders, characterized by periods of persistent low mood and anhedonia, are the third leading cause of disability worldwide [1]. Major depressive disorder (MDD) is characterized by a longitudinal trajectory of relapse and remission [2]. The economic burden of MDD is currently estimated at US $326 billion [3], with high recurrence associated with increased comorbidity burden and health care resource use [4]. Traditional assessment of MDDs is limited in its ability to detect moment-by-moment symptom changes because it relies on retrospective questionnaires completed at sporadic time points, is prone to recall bias, and is often only undertaken at the point of relapse [5]. Working toward the timely diagnosis and treatment of MDD remains an urgent priority [5].

Novel remote measurement technologies (RMTs) have the potential to become an asset for chronic disease management. Multiparametric RMT systems can provide real-time, longitudinal symptom tracking by combining active symptom reporting via smartphone apps (active RMT) with physiological and behavioral wearable sensor data (passive RMT) [6]. Continuous data can be collected on mood variability [7], sociability [8], physical activity [9], cognition [10], speech acoustics [11], and sleep [12]. Integration of RMT data into MDD care may help to more accurately assess, monitor, and predict depressive symptom trajectories, ultimately enabling personalized interventions [13]**.**

The promise of remote tracking in MDD depends almost entirely on user engagement. Engagement with mobile health (mHealth) technologies comprises the initial and sustained active use of a device [14]. High engagement with RMTs is imperative given the high-frequency data needed to identify symptom patterns and changes over time. Several systematic reviews have highlighted the heterogeneity of engagement metrics reported in remote tracking studies [15-17]. The Remote Assessment of Disease and Relapse-Major Depressive Disorder (RADAR-MDD) study is currently the largest multisite longitudinal study of a multiparametric RMT system for tracking depression [6]. The RADAR-MDD study has recently reported promising engagement, both in terms of initial recruitment rates [18] and sustained retention and data availability [19] over a 2-year follow-up of 623 participants across 3 European sites (United Kingdom, Spain, and the Netherlands). A large proportion of participants (79.8%) completed follow-up, and approximately 50% of the participants had >76% data completion for passive data streams [19].

When evaluating engagement, an understanding of the subjective experience of using RMTs should complement objective data completion statistics [17]. Subjective engagement with mHealth technologies can be understood as an experiential construct of *what it feels like* [20]. Exploring subjective engagement with RMTs provides a richer insight into system usability and perceived utility of, and satisfaction with, the technology [17]. The drivers for sustained user engagement with RMT systems, which, in contrast to typical mHealth technologies, require long periods of use for little direct rewards or intervention [21], are currently unknown.

Several studies have qualitatively explored subjective engagement with RMTs for depression. A multisite exploration of the perceived barriers and facilitators to RMT use by Simblett et al [22] informed the design of the RADAR-MDD study. Functional (technological convenience, accessibility, and intrusiveness) and nonfunctional (user cognition, perceived rewards) factors influenced patients when considering remote symptom tracking [22]. These findings have been replicated across patient and physician perspectives [23-25]. Two systematic reviews [26,27] on broader mHealth technologies for depression explored the experiences of participants’ actual use for up to 1 year. Factors such as lower symptom severity, perceived usefulness of the technology, lower privacy concerns, lack of technical issues, and access to responsive personal support were associated with enhanced motivation to engage with technologies [26,27]. A handful of studies have also suggested the beneficial effects of symptom monitoring, including increased self-awareness [28], adaptation of self-management strategies [29], and access to a “safety net” of support [30]. However, these studies typically use hypothetical scenarios or evaluate short-term system use. As a result, little is known about the subjective experience of long-term, real-world use of RMTs.

### Objective

This study aims to understand the subjective experience of long-term engagement with RMTs for monitoring depression symptoms. It uses qualitative data from the RADAR-MDD study as an example of sustained RMT use across a 2-year follow-up period. This study builds on previous qualitative work by Simblett et al [22] on perceived barriers to and facilitators of intended RMT use in depression, providing a comparison with user experiences over 2 years of sustained engagement. Our objectives were (1) to explore key themes associated with long-term RMT use and (2) to identify recommendations for future system engagement. The findings will complement the objective engagement data and provide a basis for further promotion of engagement with RMTs for symptom tracking in research and clinical practice.

## Methods

### Design

This study used a multisite longitudinal qualitative research [31] approach with thematic analysis. Semistructured interviews were conducted with participants at 3-, 12-, and 24-month time points at 3 RADAR-MDD sites: King’s College London (London, United Kingdom), Centro de Investigación Biomédica en Red (Barcelona, Spain), and Amsterdam University Medical Centre (Amsterdam, the Netherlands). The design of the interview topic guide was informed by recent work on the barriers to and facilitators of RMT use in those living with depression [16,22].

### Procedure

The RADAR-MDD study used the RADAR-base system [32] for data collection. The study active RMT smartphone app delivered fortnightly validated mood and self-esteem questionnaires and 6-weekly, high-frequency experience sampling methodology (ESM) questionnaires on current state, cognitive games, and a speech task. The study passive RMT smartphone app collected passive data on ambient noise and light, Bluetooth connection, and GPS location. Participants were provided with a wearable device, the Fitbit Charge (Fitbit Inc), measuring their step count, sleep, and physical activity. Further information on the RADAR-MDD procedure is available in the protocol paper by Matcham et al [6].

Eligibility criteria for inclusion in this study were (1) current participation in RADAR-MDD (full eligibility criteria provided in the study by Matcham et al [6]) and (2) willingness to participate in a 1:1 interview with a researcher discussing their experiences of the study. Participants provided written informed consent for the interviews as part of their RADAR-MDD study participation.

The interviews were managed by the research team lead at each site. Participants were recruited using convenience sampling at each time point to maximize data collection. Interviews were face-to-face (at the respective research site) or via telephone or video call (United Kingdom and the Netherlands only). All interviewers were female and part of the participant-facing research team. Face-to-face interviews were not conducted during the COVID-19 pandemic lockdown. Participants were reimbursed for relevant travel costs and paid per interview (£10 or €10 [US $1.2]).

The interviews were semistructured using open-ended questions, designed to elicit discussions around using the study technology in daily life (Multimedia Appendix 1). The content of each topic guide reflected the expected differences between time points. For example, the 3-month guide focused on immediate problem-solving and troubleshooting, where later interviews included data sharing.

The topic guides were translated from English into Spanish and Dutch, and interviews were conducted by native speakers at each site. The interviews lasted between 30 and 60 minutes and were conducted between February 2018 and April 2021.

### Ethics Approval

The semistructured interviews were approved by the ethics committee of RADAR-MDD [6]. Ethical approvals for conducting the study were obtained from Camberwell St Giles Research Ethics Committee (reference: 17/LO/1154) in London, from Clinical Research Ethics Committee Fundacio Sant Joan de Déu (CI: PIC-128-17) in Barcelona, and from Medische Ethische Toetsingscommissie VUms (2018.012–NL63557. 029.17) in the Netherlands.

### Data Analysis Strategy

The interviews were audio recorded and transcribed verbatim. A preliminary coding framework was developed in English based on previous findings of barriers to and facilitators of RMT use in hypothetical scenarios [22]. All sites first coded example interviews for a cross-site consistency check and a discussion on revisions to the coding framework, accounting for novel codes. Each site then proceeded to recode all interviews in the native language using NVivo software (version 12; QSR International [33]) according to the final coding framework (Multimedia Appendix 2 provides a comparison of the preliminary and final coding framework). The coding was performed by independent researchers at each site. Each site sent coded NVivo data sets to the London site, with all quotes translated into English by a third-party translator briefed on the study topic [34]. The data were stored on a secure server at the London site.

Multisite data were merged into one data set and thematic maps for 3-month, 12-month, and 24-month time points were developed by 3 researchers (KW, EDL, and PP), identifying key themes and subthemes. To align with previous longitudinal qualitative research work [31], data are presented not as a longitudinal narrative but as contributing to each theme.

## Results

### Participant Characteristics

A total of 124 interviews with 99 participants were conducted across 3 sites. Of these 124 interviews, 40 (32.2%) interviews were conducted at the 3-month time point (15/40, 38% in United Kingdom; 15/40, 38% in Spain; and 10/40, 25% in the Netherlands), 42 (33.9%) at the 12-month time point (16/42, 38% at United Kingdom; 16/42, 38% at Spain; 10/42, 24% at the Netherlands), and 42 (33.9%) at the 24-month time point (15/42, 36% at United Kingdom; 16/42, 38% at Spain; 11/42, 26% at the Netherlands). A total of 17 participants took part in an interview at 2 time points; 4 participants were interviewed across all 3 time points. Participant characteristics according to time points are shown in Table 1.

**Table 1:** Participant characteristics by interview time point.

Characteristics		Time point		
		3-month (n=40)	12-month (n=42)	24-month (n=42)
**Site, n**				
	United Kingdom	15	16	15
	Spain	15	16	16
	the Netherlands	10	10	11
Age (years), mean (SD)		44.6 (12.1)	49.4 (13.5)	51.9 (15.0)
Female, n (%)		30 (75)	32 (76)	29 (69)
**Depression severity category^a^, n (%)**				
	None	4 (10)	3 (7)	5 (12)
	Mild	7 (18)	5 (12)	5 (12)
	Moderate	10 (25)	13 (31)	7 (17)
	Severe	7 (18)	10 (24)	6 (14)
	Very severe	11 (28)	9 (21)	5 (12)
	Not reported	1 (3)	2 (5)	14 (33)
**Anxiety severity category^b^, n (%)**				
	None	7 (18)	5 (12)	7 (17)
	Mild	7 (18)	10 (24)	8 (19)
	Moderate	12 (30)	13 (31)	7 (17)
	Severe	13 (33)	12 (329)	5 (12)
	Not reported	1 (3)	2 (4)	15 (36)

^a^Measured as the Inventory of Depressive Symptomatology-Self Report total score nearest to the interview time for each participant. None=0-13, mild=14-25, moderate=26-38, severe=39-48, and very severe=49-84.

^b^Measured as the Generalized Anxiety Disorder-7 item total score nearest to the interview time for each participant. None=0-5, mild=6-10, moderate=11-15, and severe=16-21.

### Themes

This study aimed to explore the subjective experience of long-term engagement with RMTs over a 2-year follow-up period. We present our results under five themes: (1) research-related factors, (2) the utility of RMTs for self-management, (3) technology-related factors, (4) clinical factors, and (5) system amendments and additions.

### Research-Related Factors

When considering initial motivations for engaging with an RMT study, contributing toward novel research findings was the most prevalent reason for taking part. Across all time points, research team support was also a key facilitator of sustained engagement in the study.

#### Altruism and Academia

Taking part in the study was an opportunity to use personal experiences of depression to help others, to advance scientific understanding, and to “give back” to the system:

I’ve suffered with depression the whole of my adult life, I’ve obviously had a lot out of the system. If I can do anything to put back, do you see what I mean—I will.

P30, 24 months, United Kingdom

Taking part for “the future, for the people who come after me” (P8, 3 months, Spain) was a strong theme that arose in all sites when discussing reasons for enrolling in the research study. Altruistic motivations continued across later time points regardless of whether participants felt they had experienced any direct benefits:

I am actually quite proud to say that I am doing this as part of research. Some people will ask me what it is [the wearable], and I say well it is good if more people get to know about it. And for the long-term benefits, might not be for me but for other people, because it might show.

P18, 12 months, United Kingdom

With regard to the RMT aspect of the study, some mentioned that it “piqued my interest” (P37, 24 months, United Kingdom) and *“*I was very intrigued by a study that kind of has consistent monitoring” (P39, 24 months, United Kingdom). However, many participants signed up with limited knowledge of the study procedure, or of the use of RMTs for health care monitoring. Thus, a lack of prior understanding of RMTs is not a barrier to initial engagement.

Privacy was not a barrier to participants upon entering the study or throughout their participation. A key reason for this was that the research was conducted in a clinical and academic setting. In the Spanish cohort, one participant viewed the study as parallel to their clinical care:

It’s not data about, about privacy, things about you, no, it’s related to a medical condition, isn’t it? A case of depression, that’s what it’s about. So if they ask you for medical data, well, it’s normal.

P25, 24 months, Spain

Any initial privacy or data security concerns were largely alleviated by the 3-month point through conversations with the research team. At later time points, privacy was not discussed frequently.

#### Research Team Support

Support from the research team was a facilitator to continued engagement with the RMTs. This was primarily practical; at 3 months, the research team provided support on how to use the devices and study apps, which was often imperative to successful enrollment into the study:

I tried it once [the wearable] and wasn’t able to...to...put it on the phone. If it hadn’t been for [researcher name]’s help I wouldn’t have made it.

P1, 3 months, Spain

The need for practical support remained a key theme at 12 months, this time concerning technological malfunctions. Ability to contact the research team through various methods and receiving a timely reply was important. Some felt comfortable with initiating support themselves: “I didn’t need that much contact personally, I could get in contact easily, if it were necessary” (P21, 24 months, the Netherlands). Others wanted more contact, for example, more points of researcher-initiated contact, or specific contact from specialists. At-hand support was essential for continued participation:

I think it is really important to have the practical support ‘cause you don’t want to be offline or not working for long than is necessary. Otherwise it goes against the purpose of the study really.

P18, 12 months, United Kingdom

There was a consensus at all time points that the research team was approachable, patient, and reassuring, helping to alleviate technological concerns.

The research team also provided emotional support to the participants. Some participants sought comfort in the knowledge that they were being monitored as part of a study: “I liked it a lot because, jeez knowing, I felt safe, you know? Because knowing that you were there...” (P25, 24 months, Spain). Others had specific examples of receiving mental health support from the research team. One participant in the British cohort received direct signposting, which was noted in both their 12-month and 24-month interview as a crucial part of their study experience:

because of the letter from [researcher] to the GP clinic I was able to get an immediate referral, and the problem is if you’re the system it’s great, if you’re not in the system it’s difficult to get in. I couldn’t have done it on my own.

P27, 12 months, United Kingdom

### Benefits of RMTs for Self-management

Despite primarily engaging with the study for altruistic reasons, many participants experienced unexpected benefits of using RMTs for symptom monitoring during their time in the study. These comprised symptom awareness and communication, both of which were integrated into self-management of depression.

#### Symptom Monitoring and Awareness

Across all 3 time points, the most frequently reported benefit was an increase in symptom awareness. Monitoring various factors related to depression, for example, mood, sleep, and exercise, increased self-reflection, and the ability to identify patterns. For example, having access to objective sleep data provided clarification and reassurance:

I loved that [the wearable data], I found that so reassuring to just relax, of course you’ve slept and then you go ok, the next time you’re lying in bed you go I’m not ever gonna sleep again but actually you have, you’ve seen that you do I think that’s brilliant, really reassuring.

P14, 3 months, United Kingdom

Although the app did not provide feedback on symptom scores, many felt that the act of answering the questionnaires prompted them to analyze how they had been feeling:

I’m more aware of it, the questions on the questionnaire, especially those that ask how I’m feeling right now raise my awareness, I feel quite average or, I’m feeling not great, sometimes you ignore these things. And if you can take more time to think about these things...maybe I need to meditate more, I really feel self-conscious...

P10, 3 months, the Netherlands

For some, answering the questionnaires and viewing the Fitbit data simply provided an understanding of their experience of depression: “I have noticed that my answers have gotten more positive throughout the year” (P22, 24 months, the Netherlands). For others, these data directly motivated behavior changes. At 3 months, the discussion focused on the motivational effects of the Fitbit data; participants felt encouraged to complete their daily step count or achieve target physical activity “badges.” Toward the later time points, these data came to act as prompts for self-care, for example, increased exercise or relaxation:

Wearing a watch and knowing that my activity matters, you know? I mean, like the steps I take have a direct effect on my health, both physical and mental, all my activity makes me more aware of it, more conscious of it and it has also been like a driving force for me to put my batteries in sport or stress management...a habit forever, so I do not want to do without it.

P26, 24 months, Spain

This became especially apparent during the 24-month interviews, when the Fitbit data were used to monitor sleep and mood symptom changes during the COVID-19 pandemic. Disruption to usual routines during this time allowed some to reflect more than ever on the benefit of monitoring exercise:

I knew in theory, exercising and getting out and so on was good for your mental health, but over Covid, the monitor helped, and the benefit would have been even better. I think I might have been worse during Covid without it.

P36, 24 months, United Kingdom

#### Communication

At each time point, the RMT data were also used for communicating personal experiences to others. Participants used their increased understanding of their depression to inform others: “For the first time it kind of occurred to me to let me partner know when I could feel it was starting...so if you see my behaviour change or I’m unresponsive this is why” (P39, 24 months, United Kingdom).

Access to the Fitbit data also facilitated joint decision-making, both for immediate symptom management and long-term strategies:

There are also days that I don’t reach 5000 steps, which will make me think oh I haven’t done that many today...my spouse will say that too, go for another walk.

P2, 3 months, the Netherlands

#### Overall Value and Utility

There was a consensus throughout that the benefits of participating in the study outweighed the costs, of which there were relatively few. Many had not envisioned any personal benefits when enrolling as they were aware that they would not receive personalized outcomes; however, had been pleasantly surprised by the integration of RMT data into their depression self-management, as early as the 3-month time point:

I think its empowering to know more about myself to understand more so I think once I can see more what the data is from collecting from data when the other apps are working and being able to see what the data is and notice any correlations then I think that will be really valuable.

P12, 3 months, United Kingdom

### Technology-Related Factors

Experience of the technology used in the study (smartphone apps and Fitbit) was the most widely cited theme across all sites. This covered the convenience of integrating the RMTs into daily life, the usability of the technology, technological malfunctions that occurred, and the extent to which participants found the technologies intrusive.

#### Convenience

Using a mobile phone and wearing a watch were already an integral part of many participants’ daily routine. The Fitbit device, “it’s basically wearing a watch” (P7, 3 months, United Kingdom), collected data passively without the need to input information, and continual wear, syncing, and charging were integrated into the routine as early as the 3-month time point. Reminder messages across the system were useful in the process of long-term integration.

One aspect that participants found more difficult to integrate into their routine was the app questionnaires. Timing of the questionnaires was often inconvenient, for example when at work, driving, or in social situations: “Obviously I’m less likely to stop my conversation to be like oh this questionnaire, because that’s a bit rude” (P4, 3 months, United Kingdom). Frequency of the ESM questionnaires was also too high from some: “it’s impossible to have a routine with that. If you have a full-time job, it’s always a bother” (P17, 24 months, the Netherlands). The participants were rarely able to change their routine to accommodate answering the questionnaires, which sometimes caused guilt. One participant in the Spanish cohort reflected on how work affected their ability to respond to app notifications during their 2-year participation:

At the beginning it was a bit difficult because I was working, then as I was on sick leave for two years, the truth is that I’ve been able to adapt quite well. And in the end, when I went back to work again, it was a bit difficult...

P1, 24 months, Spain

#### Usability

For those who received a smartphone upon enrollment, a large technological barrier was the process of “relearning” a new operating system. This was described by some as “more difficult than anticipated” (P3, 3 months, United Kingdom), particularly during the 3-month interviews, owing to adapting to a new user interface and decreased connectivity with other devices. At 24 months, some participants had adjusted to using the new device, whereas others planned to swap back upon study completion:

No, my only peeve was that I’m an Apple user and having this bloody awful Android phone, the first thing I shall do on April 1st is take my SIM card out of the Motorola thingy.

P35, 24 months, United Kingdom

#### Technological Malfunctions

The participants reported a range of technological malfunctions that affected their participation in the study. Issues with the study apps were particularly prevalent during the 3-month interviews owing to ongoing technological challenges during the early phases of the study. These included not receiving notifications, apps crashing, apps logging out, and difficulties with rescanning QR codes. Participants sometimes had limited time or motivation to report issues to the team:

I tried opening a questionnaire I wouldn’t be able to see it, I wouldn’t be able to do it and there was no way of saying this is happening or why this is happening so maybe I should have contacted you about it but I just kind of ignored it.

P4, 3 months, United Kingdom

Issues with missing data persisted throughout the 3 time points. Participants were aware of the times when the active app had been unable to submit the completed data, or the passive app had ceased monitoring. Such malfunctions often led to anxiety or guilt that they were not “correctly” participating: “Well, yes, when it didn’t work, I became a bit nervous...” (P15, 3 months, Spain).

Participants also reported frequent missing data with the Fitbit, caused either by a syncing error or inaccurate recording. These issues caused some to question the integrity of the study: “It just didn’t work and that’s not what you expect from a research study” (P18, 24 months, the Netherlands).

A participant in the Spanish cohort reflected on how these technological malfunctions affected not only their ability to participate in the study but also their experience of being able to use the resulting data:

There is data that I have missed here, and of course I was analyzing it with me in important situations of how I was, and that I have missed them, for more than a month.

P32, 24 months, Spain

#### Intrusiveness

Generally, the concept of remote monitoring, or the use of the technologies, was not regarded as intrusive. Rather, passive data collection was noted as a preferable method because “at some point you don’t notice it. You don’t notice that you’re wearing it anymore” (P18, 24 months, the Netherlands).

However, one area that caused disruption was the wearability of the Fitbit device. Several issues associated with the Fitbit strap were reported, including skin irritation, increased sweating, and allergic reactions. Some had briefly chosen to remove the device while experiencing discomfort, whereas others had purchased straps with alternative materials. At 12 months, many reported that their strap had broken, and by 24 months, some had to apply for a full device replacement. One participant felt guilty when asking the research team for their device to be repaired:

I know that the money allocated to research programs or projects is minimal, and of course, when the strap broke or the Fitbit wouldn’t charge me and then I felt really bad because I thought “oh my God, now they have to change my Fitbit.”

P26, 24 months, Spain

Waiting for a replacement strap or device meant that participants were unable to continue to use the Fitbit for self-management:

if I was going to continue and for the others who will be continuing, it will probably begin to happen more and more depending on how much people are actually exercising with them on. It only grows, that’s the problem, in my experience with the other Fitbit, that definitely happens.

P3, 12 months, United Kingdom

### Clinical Factors

The participants were asked to reflect on whether and how they could see the RMT data being used in a clinical setting. Discussions included the extent to which participants felt comfortable sharing the data, how they envisioned clinicians using the data, and how feasible this was in the current climate.

#### Views on Data Sharing

At the 12- and 24-month time points, the participants were specifically asked to comment on data sharing with medical professionals. In general, allowing trusted clinicians to view RMT data alongside medical records was acceptable, or even essential: “let’s say my whole history, my doctor already has it, if she has it more extensive, then all the better for me.” (P30, 24 months, Spain). There was some discrepancy over whether these data should automatically be available to clinicians or mediated by the patient. Some thought that medical professionals “would be in a better position to evaluate what they needed from it than me to decide that” (P32, 24 months, United Kingdom). Others worried about interpretation of the data without context:

I suppose, [I would like to] understand what it is that is proposed to be shared, and if there’s something there that would not be appropriate at that time, because I don’t know what it is until I see it, then yes, I would like to have a choice...I would want to make sure that my health record reflects actuality rather than something that can be interpreted by people incorrectly.

P31, 24 months, United Kingdom

#### Clinical Uses of RMT Data

The participants suggested several ways in which they might expect RMT data to be beneficial in clinical care. These included (1) allowing the clinician to view the “whole picture” of individual experience, (2) allowing the clinician insight into new symptoms, (3) as a way for patients to report specific areas of concern, and finally (4) as a basis for making decisions about suitable treatment or care. Importantly, treatment decisions should be reached as a joint decision involving the clinician, the patient, and the data:

I think they could actually look at the data that’s being produced, and that could assist them in helping me to come to another decision. Like, if I was deciding that I would like to move my medication down, but they’ve got the data that says, no you’re not...but if it backs it up as well, so it can work both ways, so I think it does have those benefits.

P33, 24 months, United Kingdom

Sleep data were repeatedly cited as a data stream that would cause change in treatment. Participants from all sites provided examples of conversations with their mental health clinicians*.* One participant in the British cohort also discussed their experience of integrating the sleep data into their sleep clinic appointments:

It’s too expensive for the NHS to keep on doing [sleep tests]...I said, well, actually, I can show you any time in the last six months or so...an indication of when I’m sleeping...It helped them choose what exercises I needed to do and what therapy was required, so, yes, it was extremely helpful.

P22, 12 months, United Kingdom

Presentation of objective sleep data was seen as helpful “proof” of the participant’s recent experiences:

You can tell your GP that you sleep terribly, but of course your GP can also think that you’re just worried, but with the data it’s a fact that you can prove, so that’s nice, that you have concrete info...whether you worry or complain about it or not doesn’t matter, the facts are there.

P10, 12 months, the Netherlands

#### Current Clinical Utility of RMTs

Although the potential for RMTs in clinical care was recognized, 2 key barriers to their implementation were envisioned. First, the level of technological acceptance of medical professionals influenced participant views on the long-term utility of the data. Participants in the Spanish cohort, who were recruited through their clinical care, generally reported acceptance of the study from their clinicians: “even my psychiatrist here and in Barcelona had the same way of thinking and saw that this was very useful for me and encouraged me” (P9, 24 months, Spain).

Others described more negative experiences, often causing them to question the use of the data:

I thought it would be more relevant for my neurologist, but my neurologist wasn’t particularly interested when I told him about what I was doing in the study.

P17, 12 months, United Kingdom

Second, lack of funding, resources, and time was perceived as a major roadblock to using RMT data in appointments. This was particularly apparent in the British cohort with regard to the National Health Service. For the data to be monitored and reflected on, new procedures would need to be put in place:

I would be amazed if there was sufficient funding for that...I don’t believe that the NHS have got the resources to have people monitoring this sort of stuff.

P22, 12 months, United Kingdom

Given the perceived lack of resources to effectively use RMT data in the National Health Service, some have considered how best to come to a compromise:

I think realistically, if they had that [data] and I went to them with a problem, then I would like them to be able to use it at that point. But I don’t see it as something that they would be—so, for example, if I went to them with something and if somehow, it was a part of my NHS records, if they could access that, that might be helpful to them. But I don’t see them using it other than that really.

P32, 24 months, United Kingdom

### System Amendments and Additions

Participants discussed various changes or additions to the RMT system used in this study to further encourage long-term engagement. These included suggestions for questionnaire data collection and feedback.

#### Data Collection

Across all sites and time points, the most prevalent suggestions for changes to the study design were the content of active RMT questionnaires. Participants felt that they were frequently being asked to complete the same questions, particularly within the ESM schedule, which often prompted them to provide the same answers, for example, with regard to mood changes. This affected motivation:

At first, I was more excited about it, but as time has passed, sometimes I don’t feel much like answering since the same questions get repeated.

P19, 12 months, Spain

Some also suggested the ability to postpone questionnaires if feeling too low to complete them and the ability to provide contextual information. As early as the 3-month time point, some noted that external factors affecting their mood were not being monitored within the validated mood and self-esteem questionnaires: “I notice that when my home situation isn’t great, I also fill in the questionnaires less positively” (P5, 3 months, the Netherlands). On reflection, some would have liked to have given more information at certain points:

The answers are very closed, so you can’t really answer what you feel. You know? It’s very...it’s very up in the air.

P1, 24 months, Spain

#### Data Feedback

When asked how they might wish to view their symptom data in future use, the majority felt that this was best displayed visually through in-app graphs. Many also expressed that this would need to be accompanied by a “human explanation for what those things mean” (P3, 12 months, United Kingdom). There was a discrepancy between when these data would be best received; some only expected to receive it at the end of the study, some felt that it would be more useful in real time, whereas others were cautious that receiving data during periods of low mood would be detrimental:

If I’m well I want to see it, if I’m unwell, no. If I was reporting that I was feeling suicidal I don’t think I’d want to revisit it.

P27, 24 months, United Kingdom

Furthermore, some participants considered the potential for RMT data to provide feedback on symptom patterns and changes over time, correlations with other factors, and depressive relapse prediction. Specific examples included relationships between exercise and mood, sleep and mood, and mood and concentration: “At some point I had a burn out. I’m very curious as to how my ability to concentrate changed, and if that maybe shows on the THINC-it app” (P3, 24 months, the Netherlands).

It was generally accepted that having access to data of this nature would be useful for both self-management and integration into clinical care. Looking forward at the 24-month time point, one participant at the British site explained their hopes for the future of this field:

I think trends are really quite important for me in managing what is going on...I think one of the things I am thinking would be good to come out of this is an ability to see patterns over time and then maybe being able to use that as a predictor or, I need to do some intervention here so that I don’t end up there again if that makes sense.

P30, 24 months, United Kingdom

## Discussion

### Principal Findings

An exploration of the subjective experience of long-term engagement with RMTs for depression symptom management could prove a necessary complement to objective engagement statistics, providing insights into technology usability, user experience, and facilitators of sustained use. This study aimed to (1) explore the key themes associated with long-term RMT use and (2) identify recommendations for future engagement through longitudinal qualitative analysis at 3-month, 12-month, and 24-month time points of the RADAR-MDD study.

**Figure 1:**
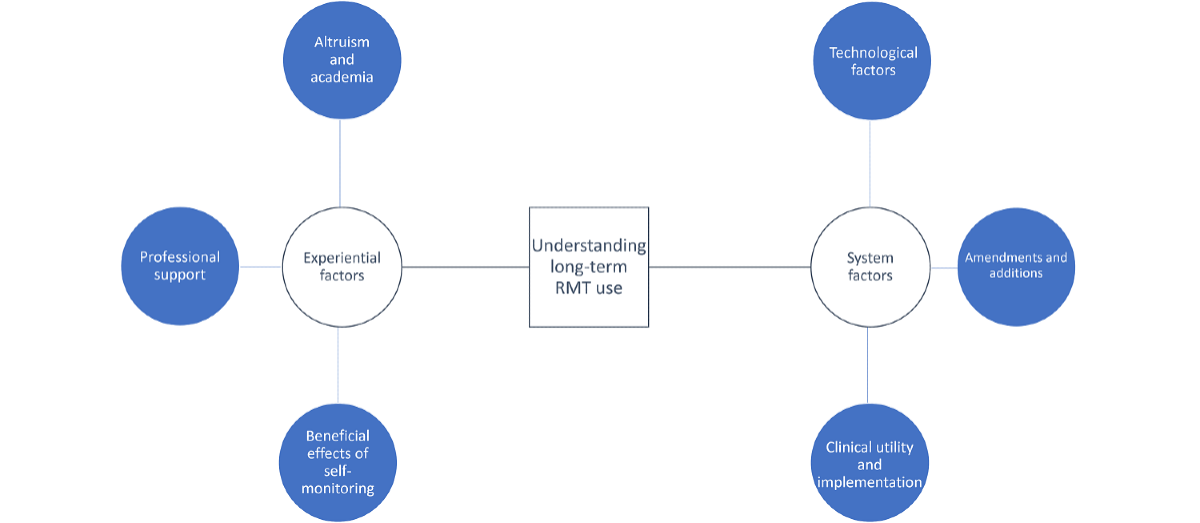
Experiential and system-related factors in the subjective experience of longitudinal remote measurement technology (RMT) use.

The themes uncovered suggest that long-term engagement with RMTs can be understood from two main perspectives: (1) experiential factors and (2) system-related factors (Figure 1). Experiential factors relate to the ways in which participants construct their experiences of engaging with RMTs for symptom monitoring. Experiential factors comprise research altruism, support from a professional team, and the benefits of using RMTs for depression management. System-related factors refer to direct engagement with the RMT systems. The factors include the usability, convenience, and intrusiveness of the technologies and the recommended system improvements for successful clinical implementation.

On the basis of these perspectives, we present a set of considerations for the promotion of engagement with RMTs for depression. Given the breadth of use cases proposed for RMTs in MDD, we focused on two areas: (1) engagement with research and (2) engagement with real-world implementation. Recommendations for engagement with future RMT research are outlined in Multimedia Appendix 3.

Although our data were derived from research participants, we believe that our findings can also be useful when considering implementation into clinical practice. Participants identified the following opportunities for RMTs in clinical care: (1) provision of feedback-informed care, (2) strengthening the therapeutic relationship, and (3) the specific clinical value of sleep monitoring. However, this potential was acknowledged with the caveat of a perceived lack of time and resources in clinical care across all 3 countries. Our findings indicate that a large difference between engagement with RMTs for research and long-term clinical engagement could be research altruism. In this study, an important facilitator of both initial and sustained engagement was the experiential factor of taking part in a novel, academic study to advance understanding and help others. To this end, participants forewent privacy concerns and initial receipt of personal benefit. They were also willing to engage despite the implementation concerns. In the absence of research altruism, Figure 1 can be used to identify further experiential facilitators that could instead be harnessed to promote engagement when RMTs become integrated into evidence-based practice. For example, clinical onboarding sessions could include a clear summary of the proposed uses and benefits of RMT data and symptom monitoring for an individual’s care. Multimedia Appendix 4 provides a set of considerations for the implementation of RMTs into clinical care based on the experiential and system-related factors identified.

**Figure 2:**
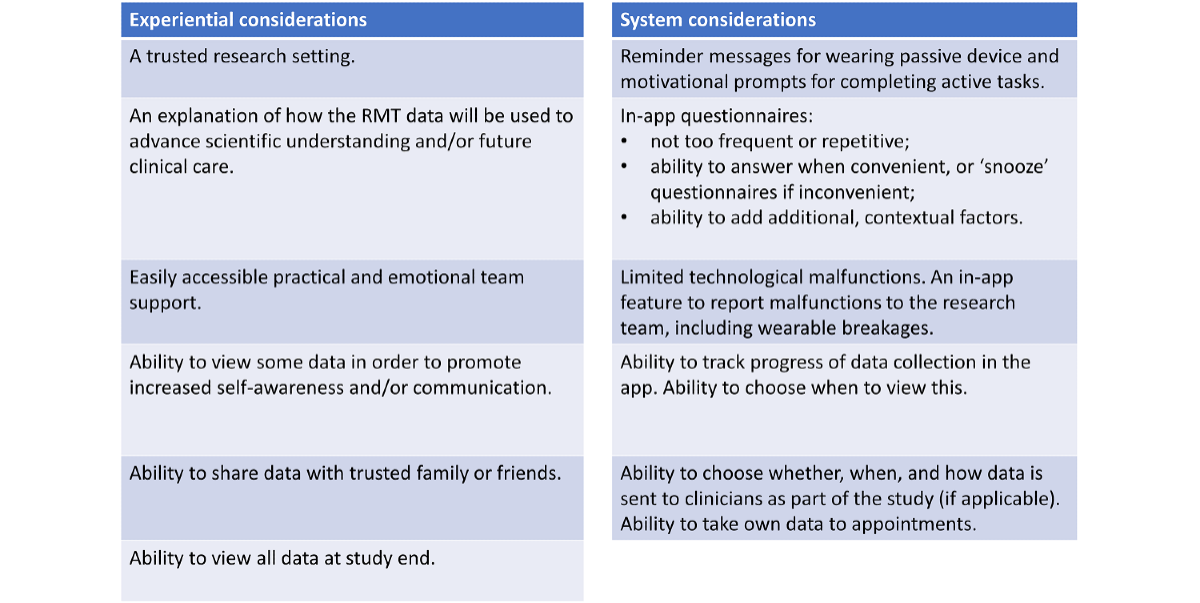
Recommendations for future remote measurement technology (RMT) use in observational research.

**Figure 3:**
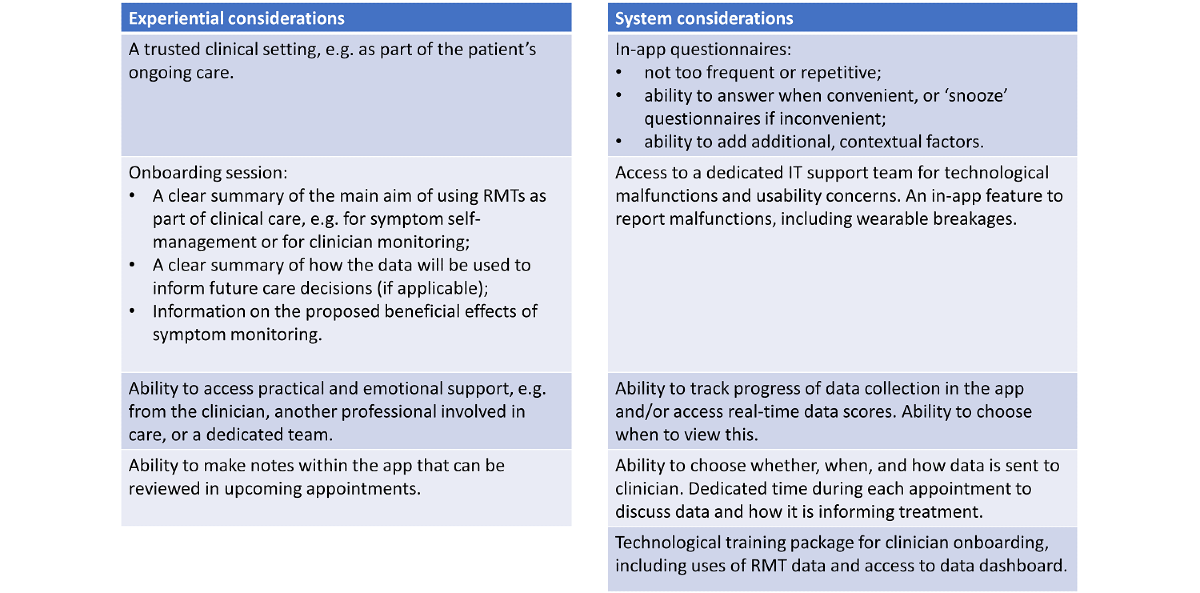
Considerations for remote measurement technology (RMT) implementation in real-world clinical settings.

### Comparison With Previous Work

This study builds on previous qualitative analyses of the barriers to and facilitators of intended RMT use for depression management. The functional and nonfunctional requirements set out by Simblett et al [22] roughly align with the system and experiential factors found here. However, a comparison of coding frameworks (Multimedia Appendix 2) revealed several differences in this study. First, nonfunctional, user-related factors such as cognition, symptom severity, and emotional resources were not acknowledged as barriers to long-term RMT engagement. Second, the overall utility of RMTs was discussed mainly in terms of benefits and rewards, and less so in terms of costs such as privacy and security. Third, studying long-term RMT use has revealed an additional layer of understanding surrounding nonfunctional requirements; experiential factors include the impact of professional support and the effects of symptom monitoring on self-awareness and communication.

When comparing our findings with those from the wider mHealth literature, technological and system-related factors remained a common theme. Borghouts et al [26] and Patel et al [27] found that lack of technical issues, flexible usability of the platform, personalization, and access to training were associated with increased long-term engagement with digital health intervention platforms. One clear difference with digital health intervention work is the focus on “a desire to actively improve one’s health” [27] as a main facilitator of initial and sustained engagement. Our work has shown that in the absence of a direct or tangible benefit, users remain willing to interact with RMTs for long periods within a research context. Experiential factors such as advancing scientific understanding and, at later periods, experiencing indirect benefits of mood tracking, seem to operate as a supplement to the user-related factors currently reported in the field.

### Strengths and Limitations

To the best of our knowledge, this is the largest study to qualitatively explore long-term RMT use for depression across multiple countries. Data collection and analyses were conducted in the native language of each country and only quotes were translated into English, aiding the transfer of meaning process [34]. However, this study has some limitations. First, where we did not anticipate any major intercountry differences in terms of attitudes toward remote mental health tracking, participants in the Spanish cohort were invited to participate by the clinicians involved in their care. This might have overinflated some themes in our analyses; for example, perceived benefits of the technologies. Second, interviews were conducted via convenience sampling of the participants who remained enrolled at each time point. This increased the risk of selection bias; those who enjoyed using the RMTs were more likely to continue to engage and as a result more likely to agree to an interview. This could explain the absence of themes relating to symptom severity or cognitive barriers present in the current work, although recent analyses have suggested that these factors did not contribute to sustained engagement in the study [35]. Convenience sampling also resulted in 21 participants completing the interviews at ≥2 time points. Preliminary sensitivity checks on a subset of this sample showed no clear signs of changes in themes over time. The data were not deemed rich enough to undertake a full, longitudinal analysis on this sample. Third, because of resource constraints, no sites undertook double coding. Fourth, data-driven themes were not explored in relation to demographic or clinical factors, as this was deemed beyond the scope of this study. Although previous work suggests that perceived usability, and actual use, of the RADAR-base system remains robust across severity of clinical characteristics [35], understanding demographic differences in subjective engagement is an important avenue for future research. Finally, the COVID-19 pandemic occurred during the study follow-up period. Given the transition to remote working and health care across all 3 countries during this time, the subjective experience of using RMTs might have been positively skewed; for example, with regard to the positive impact of the research team during social isolation. It should also be noted that the topic guide primarily asked participants to review their experience of using RMTs for this specific research project, and specific use cases for clinical implementation were not outlined by interviewers. Thus, the themes that arose from this work relate primarily to long-term engagement with RMT research, and the transferability of the findings to engagement in clinical care should be taken with caution.

### Applications for Future Research

Future work should continue to explore subjective engagement with RMTs, conceptualized in terms of both experiential and system-related factors. Where system-related factors often represent clear recommendations for technological improvements, understanding the experiential effects of engaging with RMTs is a novel finding that could prove fundamental in promoting future engagement. A recent systematic review [17] found that 5 studies have begun to explore the correlational relationship between objective and subjective engagement with RMTs. Higher daily assessment counts from an active RMT app were correlated with increased app satisfaction ratings at 3-month and 6-month time points [36,37]. Understanding the link between experiential factors, such as increased self-awareness, and objective engagement could bolster this field further.

Our findings explore the initial and sustained engagement with RMTs for depression symptom monitoring in a research setting. The next step would be to replicate this work in a clinical setting. Recent qualitative analyses have reported positive views from patients and clinicians on the potential for implementation of RMT into psychological services [38]. This paper provides considerations for adapting RMT systems for use in clinical settings and a framework for continuing to analyze the subjective experience of long-term clinical engagement to allow for further iterations.

### Conclusions

This study aimed to understand the subjective experience of long-term engagement with RMTs for depression symptom monitoring as a complement to the high rates of objective engagement observed in the RADAR-MDD study. Key experiential and system-related themes associated with long-term RMT use were identified along with a set of recommendations and considerations for promoting future system use in both research and clinical settings. Further understanding of the construction of the “experience” of using RMTs will be key to promoting long-term engagement in clinical care and depression management in comparison with general mHealth interventions that offer immediate or tangible rewards. In the wake of the rapid expansion of this field, we urge professionals to continue monitoring the subjective experience of RMT engagement to maximize the potential of remote monitoring as both a method for data collection and a tool for symptom management.

## Appendix

Multimedia Appendix 1 Main interview questions at 3-month, 12-month, and 24-month follow-up time points.

Multimedia Appendix 2 Preliminary and final codes in the coding framework.

Multimedia Appendix 3 Recommendations for future remote measurement technology (RMT) use in observational research.

Multimedia Appendix 4 Considerations for remote measurement technology (RMT) implementation in real-world clinical settings.

## Abbreviations

ESM: experience sampling methodology
MDD: major depressive disorder
mHealth: mobile health
RADAR-MDD: Remote Assessment of Disease and Relapse-Major Depressive Disorder
RMT: remote measurement technology

## Data Availability

The data supporting the findings of this study are available from the corresponding author upon reasonable request.
