# Supplementary material for: Understanding the Subjective Experience of Long-term Remote Measurement Technology Use for Symptom Tracking in People With Depression: Multisite Longitudinal Qualitative Analysis
Source: JMIR Hum Factors. 2023 Jan 26;10:e39479. doi: 10.2196/39479 (PMC9945920; doi:10.2196/39479)
Supplement: Multimedia Appendix 1 [file humanfactors_v10i1e39479_app1.docx]

Appendix 1. Main interview questions at 3-month, 12-month and 24-month follow-up timepoints.

| **3-month interview** | **12-month interview** | **24-month interview** |
| --- | --- | --- |
| **Participation experience** | **Participation experience** | **Participation experience** |
| How are you finding the experience of participating? | How are you finding the experience of participating? | What motivated you to volunteer for the study? Now that you have finished with the study, how did you find the overall experience? Were you surprised by anything as you participated in this study? Were there any unexpected experiences or outcomes? |
| **Enrolling in the study/support** | **Enrolling in the study/support** | **Enrolling in the study/support** |
| Did you have any concerns coming into the study? | More of a focus on practical support | Was there anything you wish you had known at the beginning of the study? What made you feel that way? |
| **Working the study into daily life** | **Working the study into daily life** | **Working the study into daily life** |
| Has taking part in this study fit with your usual routine? | Does your involvement in the study ever change the way you manage your health? | What could have made it easier for you to take part in this study? |
| **Experience with the apps/devices** | **Experience with the apps/devices** | **Experience with the apps/devices** |
| Were you familiar with the smartphones and/or the wearable device before you started using them as part of this study? | How does your experience in this study compare to your past experiences with this type of technology? | How did your experience in this study compare to your past experiences with this type of technology? |
| **Experience with the study surveys** | **Experience with the study surveys** | **Experience with the study surveys** |
| How do you find the study’s applications and mobile surveys to use? | Would it be useful to receive information about the data collected throughout the study? | Would it be useful to receive information about the data collected throughout the study? |
| **Experience with wearable devices** | **Experience with wearable devices** | **Experience with wearable devices** |
| How do you find the appearance and comfort of the device? | How did you feel about the data being collected passively, without you needing to interact with the device? | How did you feel about the data being collected passively, without you needing to interact with the device? |
| **Health anxiety and devices** | **Health anxiety and devices** | **Health anxiety and devices** |
| Has there been anything about the mobile device that has made you uncomfortable (concerned, worried)? Please describe. | Has there been anything about the mobile device that has made you uncomfortable (concerned, worried)? Please describe. | Has there been anything about the mobile device that has made you uncomfortable (concerned, worried)? Please describe. |
| **-** | **Decision making** | **Decision making** |
|  | Has the device (not the study but specifically the device) contributed to any decision-making you have made about your health? | Has the device (not the study but specifically the device) contributed to any decision-making you have made about your health? |
| **-** | **Data privacy & sharing** | **Data privacy & sharing** |
|  | How would you feel about the information collected during the study going to your GP or healthcare provider? What would you like your GP or healthcare provider to do with the information? | How would you feel about the information collected during the study going to your GP or healthcare provider? What would you like your GP or healthcare provider to do with the information? |
| **Closing & improvements** | **Closing & improvements** | **Closing & improvements** |
| Do you find that the benefits of taking part outweigh the costs of taking part? Or the other way round that the costs outweigh the benefits? | Do you find that the benefits of taking part outweigh the costs of taking part? Or the other way round that the costs outweigh the benefits? | Do you find that the benefits of taking part outweigh the costs of taking part? Or the other way round that the costs outweigh the benefits? |
