# Supplementary material for: Understanding the Subjective Experience of Long-term Remote Measurement Technology Use for Symptom Tracking in People With Depression: Multisite Longitudinal Qualitative Analysis
Source: JMIR Hum Factors. 2023 Jan 26;10:e39479. doi: 10.2196/39479 (PMC9945920; doi:10.2196/39479)
Supplement: Multimedia Appendix 2 [file humanfactors_v10i1e39479_app2.docx]

Appendix 2. Preliminary and final codes in the coding framework.

|  | *Name* |
| --- | --- |
| **Preliminary coding framework** | **Final coding framework** |
| **1. Uses of mHealth** | **1. Uses of mHealth** |
| 1.1 Self-Monitoring | 1.1 Self-Monitoring |
| 1.2 Clinical Prediction | 1.2 Clinical Prediction |
| 1.3 Intervention Delivery | 1.3 Intervention Delivery |
| 1.4 Communication Aid | 1.4 Communication Aid |
| 1.5 Other | 1.5 Other |
| **2. Service-User Related Barriers and Facilitators** | **2. Service-User Related Factors** |
| 2.1 Perceived Rewards | 2.1 Perceived Rewards |
| 2.2 Perceived Costs | 2.2 Perceived Costs |
| 2.3 Overall Value | 2.3 Overall Value |
| 2.4 Technology Acceptance (self) | 2.4 Technology Acceptance (self) |
| 2.5 Other | 2.5 Technology Literacy |
|  | 2.6 Other |
| **3. Technology-Related Barriers and Facilitators** | **3. Technology-Related Factors** |
| 3.1 Convenience | 3.1 Convenience |
| 3.2 Accessibility | 3.2 Accessibility |
| 3.3 Usability | 3.3 Usability |
| 3.4 Intrusiveness | 3.4 Intrusiveness |
| 3.5 Other | 3.5 Malfunctions and Technical Issues |
|  | 3.6 Other |
| **4. Health-Related Barriers and Facilitators** | **4. Health-Related Factors** |
| 4.1 Symptom Intensity or Severity | 4.1 Symptom Intensity or Severity |
| 4.2 Cognitive Ability | 4.2 Cognitive Ability |
| 4.3 Awareness | 4.3 Awareness |
| 4.4 Emotional Resources | 4.4 Emotional Resources |
| 4.5 Physical Ability | 4.5 Physical Ability |
| 4.6 Other | 4.6 Other |
| **5. System-Related Barriers and Facilitators** | **5. System-Related Factors** |
| 5.1 Support | 5.1 Support |
| 5.2 Technology Acceptance (others) | 5.2 Technology Acceptance (others) |
| 5.3 Data Sharing | 5.3 Data Sharing |
| 5.4 Other | 5.4 Recommended Design Features and Functions |
|  | 5.5 Other |
| **6. Other** | **6. Research-Related Factors** |
|  | 6.1 Support |
|  | 6.2 Methodology and Design |
|  | 6.3 Altruistic Engagement |
|  | 6.4 Other |
|  | **7. Other** |
|  | 7.1 Coronavirus |
|  |  |
|  |  |

NB: Red text highlights the additional themes and sub-themes that were uncovered in the final coding framework.
