# Supplementary material for: Understanding the Subjective Experience of Long-term Remote Measurement Technology Use for Symptom Tracking in People With Depression: Multisite Longitudinal Qualitative Analysis
Source: JMIR Hum Factors. 2023 Jan 26;10:e39479. doi: 10.2196/39479 (PMC9945920; doi:10.2196/39479)

Multimedia Appendix 3

Recommendations for future remote measurement technology (RMT) use in observational research.
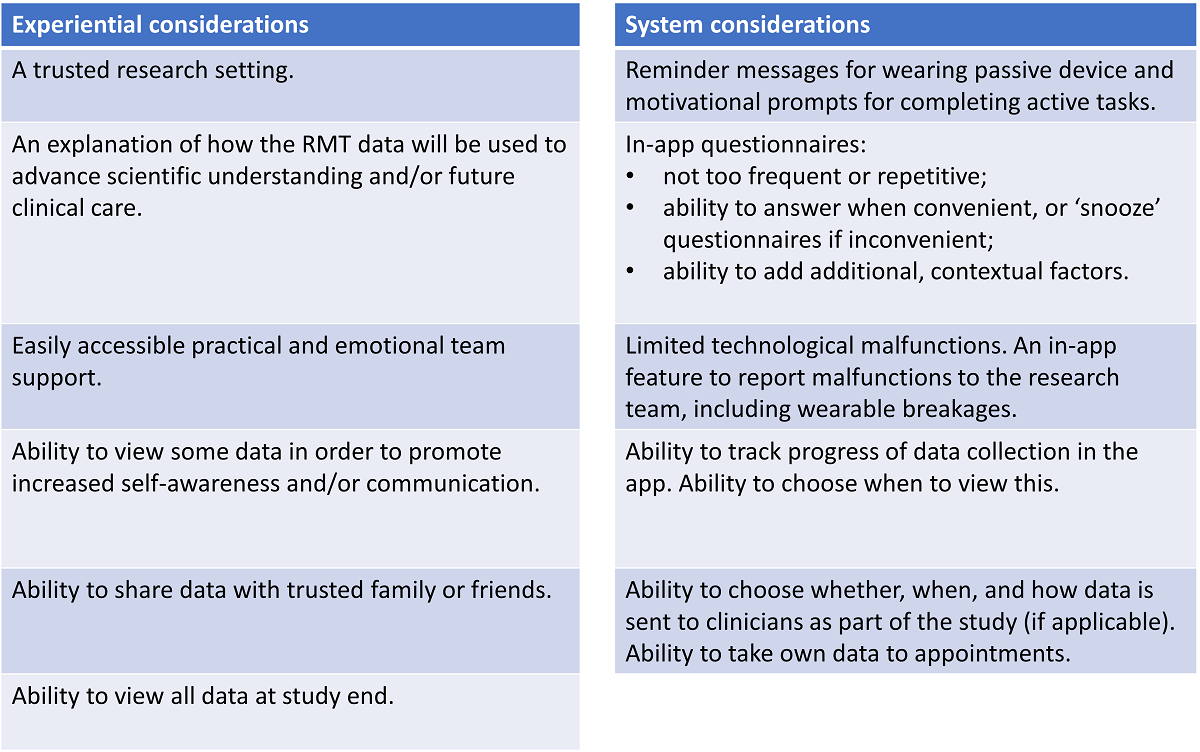

Supplement: Multimedia Appendix 3 [file humanfactors_v10i1e39479_app3.docx]
